# Prevalence and determinants of scabies among school-age children in Central Armachiho district, Northwest, Ethiopia

**DOI:** 10.1371/journal.pone.0269918

**Published:** 2022-06-14

**Authors:** Bisrat Misganaw, Solomon Gedlu Nigatu, Gebremedhin Necho Gebrie, Anteneh Ayelign Kibret

**Affiliations:** 1 Department of Epidemiology and Biostatistics, University of Gondar Institute of Public Health, Gondar, Ethiopia; 2 Tach Armachio Health Office, Amhara, Ethiopia; 3 Department of Human Anatomy, School of Medicine, University of Gondar College of Medicine and Health Science, Gondar, Ethiopia; King Faisal University, SAUDI ARABIA

## Abstract

**Background:**

Scabies is a major global public health issue that might affect people from all socioeconomic levels. Globally, scabies affects more than 200 million people at any time. It remains one of the commonest skin diseases seen in developing countries including Ethiopia. Therefore, this study aimed to assess the prevalence and determinants of scabies among school-age children in Central Armachiho district, Northwest Ethiopia.

**Methods:**

A community-based cross-sectional study was conducted from August to September 2020. A multi-stage sampling technique was used to select 850 study populations. Data was checked for its completeness, coded, and entered by using EPI-INFO version 7 and exported to the SPSS version 20 for analysis. A Binary logistic regression model was fitted to identify the determinants of scabies. Crude odds ratio (COR) and adjusted odds ratio (AOR) with 95% CI were used as measurements for the associations. P-values <0.005 were considered significant.

**Result:**

Prevalence of scabies among the 850 participants studied was 10.82% (95% CI: 8.7–12.9). Contact history with confirmed scabies patient (AOR = 5.28,95% CI: 2.96–9.44), child not attending school (AOR = 3.08, 95% CI;1.45–6.54), rarely changing clothes (AOR = 2.43,95% CI: 1.27–4.62), sleeping on the floor (AOR = 4.11, 95% CI:1.95–8.67), bed sharing; (AOR = 3.38, 95% CI:2.86–6.15), rarely washing cloth: (AOR = 5.08,95% CI:2.75–9.36), living with internally displaced people; (AOR,95% CI: 3.47 (1.30–9.24) and using only water to wash hands; (AOR = 3.18,95% CI:1.74–5.80) had a statistically significant association with scabies infestation among school-age children.

**Conclusion:**

The current study found nearly one out of ten school-age children had scabies. Not attended school, contact history with confirmed scabies patient, not washing cloth, infrequent changing clothes, bedding sharing, sleeping on the floor, living with internally displaced people, and only using water for handwashing practice were the independent predictors for the occurrence of scabies. Health education better to given to the parents or caregivers about the washing of clothing, changing clothes at least once per week, and avoid physical contact with known scabies cases.

## Background

Scabies is a contagious skin infestation caused by the obligatory human parasite mite *Sarcoptes scabiei* penetrating the skin [1, 2]. Even though scabies is more common in tropical and humid regions, it is neglected by health professionals and researchers. Globally, it is estimated that scabies affects more than 200 million people at any time [3]. The prevalence of scabies ranges from 0.3%-71%, worldwide [4] and accounts for 0.21% of disability-adjusted life-years (DALYs) [5]. Different epidemiological studies were conducted in some part of Africa to indicate the prevalence of scabies, such as in Gambia (15.9%), and Cameroon (17.8%) [6, 7]. In Ethiopia, scabies is also common, especially during natural or manmade disasters such as flooding, drought, civil war and conflict, poor water supply and sanitation, and overcrowding living conditions [8]. A systematic review and meta-analysis study was conducted in Ethiopia to determine the a prevalence of scabies in all age groups and was found to be 14.5% [9]. Besides, other studies conducted in Arbaminch, Dabat, and Amhara drought-affected areas revealed that the prevalence of scabies infestation is 16.4%, 9.3%, and 33.7% respectively [8, 10, 11]. The characteristic symptoms of a scabies infestations include superficial burrows, intense itching especially at night, a generalized rash that may affect much of the body or be limited to cold sites including the wrist, elbow, armpit, nipple, genitalia, waist, buttocks, and the area between the fingers [12]. Scabies can lead to opportunistic bacterial infections ranging from simple skin infection to severe systemic disease like septicemia, renal failure, and possibly rheumatic heart disease [13, 14]. Besides, scabies also causes a huge public health burden including stigma, social isolation, absenteeism, sleeping disturbance, and also has a 5–10% fatality rate per year [15, 16]. Scabies and its complication impose a considerable economic burden on individuals, families, communities, and health systems [17, 18].

Even though scabies is easily treatable disease, it is a major public health issue and continuous as neglected tropical disease, particularly in low-resource areas. Ethiopia is one of the countries affected by the scabies outbreaks. The distribution of scabies outbreak varied across regions and districts of Ethiopia, especially in the places where poor access to water sources, infrastructures, and food security, the burden of scabies is high [19]. It is also a major problem among Ethiopian primary school children. Although there were some studies in different parts of Ethiopia [20–24], there is a scarcity of evidence regarding scabies prevalence and associated risk factors among schoolchildren in our study area. Therefore, this study aimed to assess the prevalence and determinants of scabies among school-age children in Central Armachiho district, Northwest, Ethiopia.

## Methods

### Study design and setting

A community-based cross-sectional study was conducted among school-age children in Central Armachiho district, Northwest Ethiopia from August to September 2020 G.C. Ethiopia is administratively divided in to four level; regions, zones, woredas (districts), and finally kebele. Central Armachiho district is located 832 kilometers North-West of Addis Ababa & 288 kilometers from Bahir Dar, Capital City of Amhara National Regional State. The district has a total population of 84,600 (40,692 male and 43,908 female) with 24,610 24,610 being school-age children, based on the 2013 Ethiopian fiscal year (EFY) Woreda population projection. Within the Woreda, there are 4 health centers and 15 health posts serve for 15 Kebles’ population.

### Population and sample size determination

The source population for this study was all school-age children who live in the Central Armachiho district. The study population was all school-age children living in the selected “Gotts” of Kebele in the Central Armachiho district. School-age children (5–14 years old) lived in the district during the study period included in the study. However, School-age children who have other skin diseases confirmed by a health professional were excluded from the study. The sample size was determined using a single population proportion formula, by considering the following assumptions; 95% confidence interval, 2.5% margin of error, 10% non-respondent, A design effect of 1.5, the expected proportion of scabies was considered to be 9.3% from the study conducted in Dabat [11]. The final sample size was found to be 857. Study participants were selected using a multistage cluster sampling technique. The primary sampling unit was the kebele (the smallest administrative unit in the country), and there are 14 kebeles in the district. Out of the total kebeles, five (Masero, Sanki, Felfel, Befegn, and Jansuma) were selected by a simple random sampling method. The secondary sampling unit was “Gott” (village) and 20% of “Got” in the five kebeles were selected using a simple random sampling technique. Every school-age child residence of the selected “Gott” was the final sample unit. Based on the proportion allocation household in each selected “Gott” was selected using a systematic random sampling technique. If more than one school-age child presented in the household a single child was selected by a lottery technique.

### Variables and data collection procedures

The outcome variable for this study was scabies status, which was diagnosed based on history and physical examination. In this study scabies means the presence of persistent pruritic rash with itching increasing at night which are notified at least at two specific body sites (on the wrist, sides and web spaces of the fingers, the axillae, periareolar, per umbilical, genitalia area, abdomen, and buttock areas) with or without a history of pruritus in the close entourage [25]. The first group of factors assessed was socio-demographic and family characteristics; age and sex of the child, area of residence, education of mothers, education of father, education of children, occupation of mothers, occupation of father, wealth index of the household, family size, religion, ethnicity, marital status of household head (HH), and living with internally displaced people. The second group of factors was scabies-related knowledge among mothers of school-age children. In addition to this, environmental, personal hygiene, and sanitation factors were also assessed (S1 Table). Children in the age group of 5–14 years old were considered school-age [26]. Knowledge related to scabies for mothers of school-age children was assessed as participants were asked to answer 14 knowledge-related Yes/No questions about the nature, ways of transmission, prevention, and control measures of scabies. If they successfully answered the median and above the number of questions (9 to 14 out of 14), they were judged to have "good knowledge," and otherwise, they were considered to have "poor knowledge". Drinking water sources were classified as "protected" if the community obtained water from protected springs, wells, or public taps, and "unprotected" if the community obtained water from a river, unprotected springs, or unprotected wells [27]. Hand washing practice is considered "good" if children wash their hands with soap before eating, after defecation, after meals, before food preparation, after going to the bathroom, and after handling trash or animals. The overcrowding index was calculated by dividing the number of usual residents in a house by the number of bedrooms in the house. If it is more than 1.5 overcrowded and if it is less than or equal to 1.5 not overcrowded [28]. Infrequent washing clothes means washing clothes less than once per week in the past month and infrequent changing clothes means changing clothes less than once per week in the past month [29].

The interviewer-administered questionnaire was adopted from different works of literature that were completed by trained four public health officers using the Amharic version questionnaire. The questionnaire was written in English first, then translated into Amharic and back to English, with the consistency confirmed. The data quality was ensured by providing one-day training to the data collectors. To evaluate the consistency and clarity of the developed questionnaire, a pretest was carried out using 5% of the sample size in Tach Armachiho wereda. Hence, corrections and modifications were made to the questionnaire accordingly.

### Data processing and analysis

The survey data was entered using EPI-INFO version 7 and analyzed using STATA 14 software. Descriptive statistics were used and presented using texts, graphs, and tables. Multivariable logistic regression analysis was fitted, and the corresponding adjusted odds ratio (AOR) and 95% CI were used to identify factors associated with scabies. A p- value < 0.05 was used to characterize statistically significant results. The Hosmer -Lemeshow goodness-of- fit statistic was used to assess whether the necessary assumptions for the application of multiple logistic regression are fulfilled. For this study, a p-value was found to be 0.89, implying that the model is good fit.

### Ethical issues

Ethical clearance was obtained from the Institutional Ethical Review Board of the Institute of Public Health, College of Medicine and Health Sciences, University of Gondar (IRC of UOG; Ref. /No/ IPH /1092/2020). The support and permission letter were obtained from Biostatistics and Epidemiology Department and was given to the Central Armachiho Woreda. Mothers/caregivers were informed about the purpose, objectives, and their right to and not to participate in the study. Oral informed consent was obtained from mothers/caregivers. To keep confidentiality, respondents’ names and other personal identifiers were not included in the questionnaire. Data collected were password-protected.

## Result

### Socio-demographic and family characteristics of the participates

Our total sample size was 857, unfortunately the mother/ caregiver of seven of study participants didn’t give the required information due to, some of respondents were ill and some of them absent during data collection period. Finally, a total of 850 study participants were included in this study with a response rate of 98.04%. The mean age of the school-age children was 9.54 years with a 2.54 standard deviation. Majority of the children examined were females (54.9%), from Masero (61.3%), resident in rural areas (60.4%). Of the participants 821 (96.59%) were living in overcrowding families. Of mothers and fathers of school-age children, 520 (61.20%) and 489 (57.53%) were not educated respectively. More than of half of (59.06%) of mothers were housewife and (61.35%) of fathers of school-age children were farmers (Table 1).

**Table 1:** Socio demographic characteristics of the respondents, Central Armachiho district, Northwest Ethiopia, 2020, (n = 850).

Variable	Frequency (n = 850)	Percent (%)
**Sex of school age children**		
Male	383	45.06
Female	467	54.94
**Kebele**		
Befegn	89	10.47
Felfel	69	8.12
Jansuma	106	12.47
Masero	521	61.29
Sanki	65	7.65
**Age(year)**		
5–9	430	50.59
10–14	420	49.41
**Residency**		
Urban	337	39.65
Rural	513	60.35
**Mothers’ education status**		
Not educated	520	61.20
Educated	330	38.80
**Fathers’ education status**		
Not educated	489	57.53
Educated	361	42.47
**Education status of children**		
Not attended	159	15.65
Grade 1–4	475	57.88
Grade 5–8	216	26.47
**Occupation of mothers**		
Housewife	502	59.06
Farmer	63	7.41
Governmental work	48	5.65
Daily labor	19	2.24
Non-governmental	24	2.82
Merchant	191	22.47
Others	3	0.35
**Occupations of fathers**		
Non-governmental	16	1.88
Farmer	530	62.35
Governmental work	99	11.65
Merchant	194	22.82
Others	11	1.29
**Marital status of HH**		
Married	834	98.12
Others	26	3.06
Religion of HH		
Orthodox	819	96.35
Muslims	30	3.53
Others	1	0.12
**Ethnicity of HH**		
Amhara	842	99.06
Others*	8	0.94
Living with internally displaced people		
No	817	96.24
Yes	33	3.76
Wealth index for urban/rural		
Lowest	242	28.47
The second	144	16.94
Middle	175	20.59
The fourth	115	13.53
The highest	174	20.47
**Family size to bed rooms ratio**		
≤15	29	3.41
>15	821	96.59

Others*: Tigray, Oromia, and Afar

### Scabies-related knowledge among mothers/caregivers

Of the total parents, 795 (93.53%) ever heard about scabies and 193 (22.71%) knew about scabies. Among the school-age children’s mothers, 684 (89.88%) knew the mode of scabies of transmission, and 773 (90.94%) identified prevention strategies. Of the children’s mothers, 704 (82.82%) had good knowledge about scabies.

### Environmental related characteristics

One-fourth (216) of the parents of the school-age children did not get drinking water from protected water sources. Out of the school-age children, 76 (4.12%) slept on the floor, and one-fifth (178) had a contact history with known scabies infested individual (Table 2).

**Table 2:** Environmental characteristics of the school age children in Central Armachiho district, Northwest Ethiopia, 2020.

Variable	Frequency(n = 850)	Percent (%)
**Place of sleep**		
On the floor	76	4.12
On bed	774	95.88
**Time taken to Health facility**		
Near (<1hours)	671	78.94
Far (≥1 hours)	179	21.06
**Ever had contact with scabies case**		
No	672	79.12
Yes	178	20.88
**Water source**		
Un protected water	216	25.41
Protected water	634	74.59
**Time taken to source of drinking water**		
Near (<1/2hours)	671	78.94
Far (≥1/2 hours)	169	19.88
**Daily requirement of water for household**		
Not enough (<50 liter)	666	78.35
Enough (50 liter)	184	21.65
**Traveling history of family to scabies endemic area**		
No	836	98.35
Yes	14	1.65

### Personal hygiene and sanitation-related characteristics

Out of the total study participants, 260 (30.24%) took a bath and 195 (22.94%) washed clothes infrequently. Of the participants, 601 (71.65%) used water and soap/ash for hand washing and 142 (16.7%) did not wash their clothes (Table 3).

**Table 3:** Personal hygiene and sanitation characteristics of the school age children in Central Armachiho district, Northwest Ethiopia, 2020.

Variables	Frequency(n = 850)	Percent (%)
**Frequency of bathing**		
Infrequent	260	30.24
Frequent	590	69.76
**Wash wearing of your cloth**		
No	142	16.7
Yes	708	708
**Frequency of washing cloth**		
Infrequent	195	22.94
Frequent	648	76.24
Do not	7	0.82
**Sleeping with family members**		
Alone	92	10.82
With others	758	89.18
**Bedding/Cloth sharing**		
No	561	73.06
Yes	289	26.94
**Frequency of changing cloth**		
Infrequent	287	33.76
Frequent	563	66.24
**Wash hands at critical time**		
No	41	4.82
Yes	809	95.18
**Frequently used detergents**		
Only water	241	28.35
Wash with detergents	609	71.65
**Wearing cloths such as T-shirt, pants, etc.) of who had scabies**		
No	835	98.24
Yes	15	1.76

### Prevalence of scabies among school-age children

**Fig 1:**
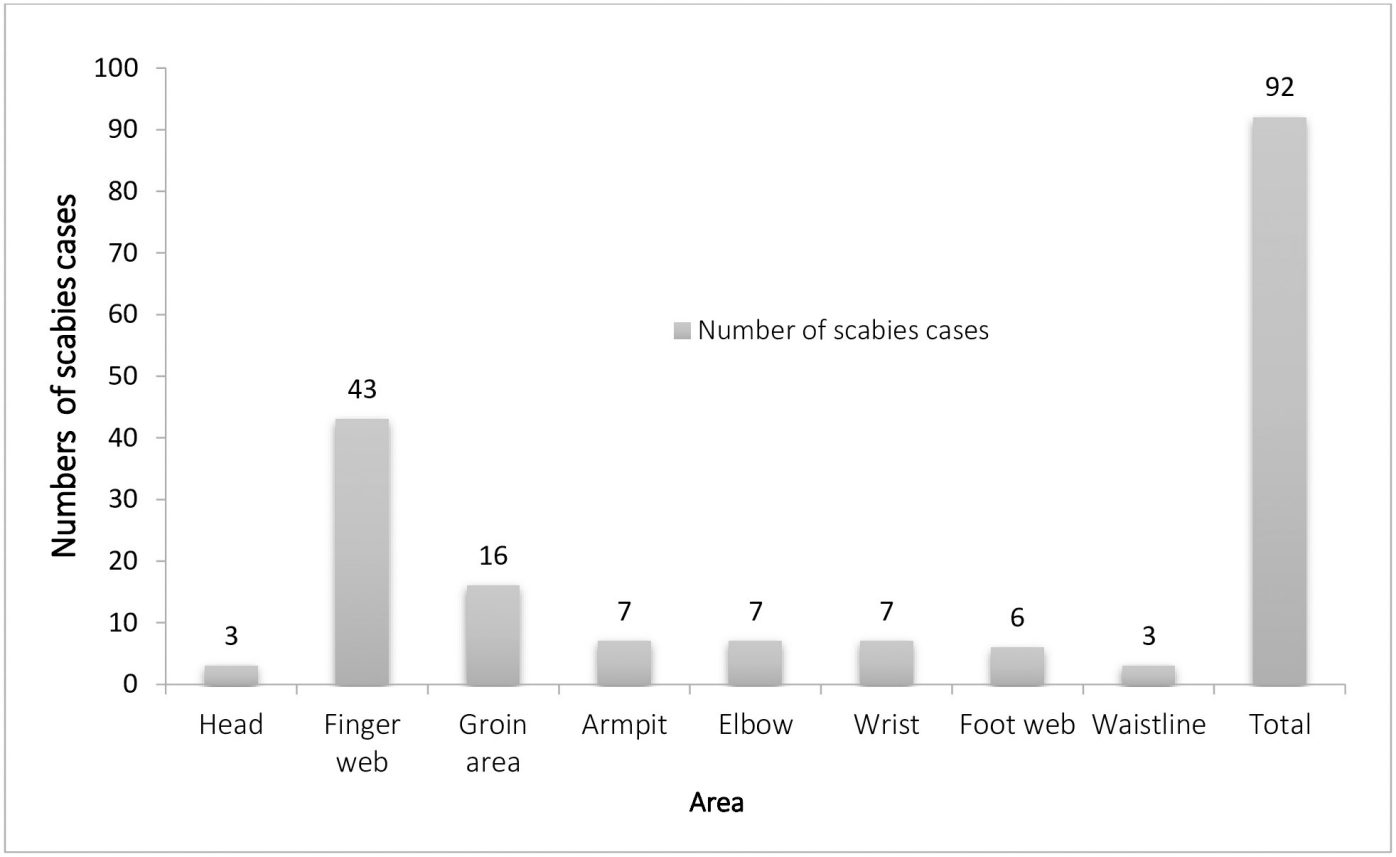
Number of scabies cases by area of infestation among school age children in Central Amarchiho district North West Ethiopia, 2020.

The overall prevalence of scabies among school-age children was 10.82% (95% CI: 8.7, 12.9). Of the 92 cases of scabies, 61 (66.31%), 10(10.86%), 12(13.05%), 7(7.61%), and 2(2.17%) were found in Masero, Felfel, Jansuma, Befegn, and Sanki Keble respectively. More than half of scabies cases were occurred in females, 55(59.78%) and 48 (52.17%) cases happened in the age of 5–9 years. The most common locations of scabies lesions were the web spaces between the fingers 43(46.74%) (Fig 1).

### Determinants of scabies

From the multivariable logistic regression analysis, educational status of school-age children, sleeping place, bedding/cloth sharing, frequency of changing cloth, washing cloth, history of contact with scabies, frequency of handwashing, and living with internally displaced people from other areas were statistically significant determinants of scabies among school-age children in our study area. The odds of having scabies were significantly higher among school-age children who had a history of contact with scabies compared with those without a history of contact (AOR = 5.28,95% CI: 2.96, 9.44), higher among children who slept on the floor compared with those who slept on a bed (AOR = 4.11, 95% CI: 1.95, 8.67). The odds of having scabies were higher among school-age children who share clothes compared to those who didn’t share clothes (AOR = 3.38, 95% CI: 2.86, 6.15). The school-age children who used only water for handwashing were more likely to be infested by scabies than the school-age children who washed their hands with water and soap/ash (AOR = 3.18, 95% CI: 1.74, 5.80). The odds of having scabies were higher among school-age children who changed clothes infrequently than those who had changed clothes at least once a week (frequently) (AOR = 2.43, 95% CI: 1.27–4.62). The odds of being infested by scabies among school-age children were higher in those who did not wash clothes than in those who washed clothes regularly (AOR = 5.08, 95% CI: 2.75–9.36). School-age children who lived with internally displaced people were more likely to be infested with scabies than their counterparts (AOR = 3.47,95%CI: 1.30–9.24). The odds of having scabies were significantly higher among school-age children who had not attended school compared to those at 5–8 grade level (AOR = 3.08,95%CI;1.45–6.54) (Table 4).

**Table 4:** Binary and multiple logistic regression analysis of determinants for scabies among school age children in Central Armachiho district, North West Ethiopia 2020.

Variable	Having scabies		COR (95% CI)	AOR (95% CI)
	Yes	No		
**Education status of school age children**				
Not attended school	71	88	2.73(1.56,4.79)	**3.08(1.45,6.54)** **
Grade 1–4	197	278	0.57(0.32,0.99)	0.65(0.32,1.32)
Grade 5–8	56	160	1	
**Sleeping place**				
On the floor	28	48	6.47(3.8,11.03)	**4.11(1.95,8.67)** ***
On bed	64	710	1	
**Bedding sharing**				
No	60	229	1	
yes	32	529	4.33(2.75,6.84)	**3.38(1.86,6.15)** ***
**Frequency of changing cloth**				
Infrequent	54	233	3.20(2.06,4.99)	**2.43(1.27,4.62)** **
Frequent	38	525	1	
**Frequency of bathing**				
Infrequent	535	223	1.61(1.03,2.52)	0.85(0.47,1.59)
Frequent	55	37	1	
**Washing cloth**				
No	48	94	7.7(4.85,12.24)	**5.07(2.75,9.36)** ***
Yes	44	664	1	
**Frequency of washing cloth**				
Infrequent	33	162	2.07(1.31,2.29)	1.05(0.52,2.11)
Frequent	58	590	1	
**Contact history of scabies the last two weeks**				
No	639	33	1	
Yes	119	59	9.6(6.09,15.34)	**5.28(2.96,9.44)** ***
**Frequently used detergent**				
Only water	51	190	3.72(2.39,5.80)	**3.18(1.74,5.80)** ***
With detergent	41	568	1	
**Lived with IDP**				
No	12	21	1	
yes	80	737	5.26(2.50,11.0)	**3.47(1.30,9.24)** *

NB.

* significant at p≤0.05,

** significant at p≤0.01,

*** significant at p≤0.001,

1 = Reference, Hosmer–Lemshow goodness-of-fit with p = 0.13, COR = Crude Odds Ratio, AOR = Adjusted Odds ratio, CI = Confidence interval

## Discussion

Epidemiological studies about the burden and associated factors of scabies provide valuable information about the disease and serve as a basis for methods of prevention, control, and management services. Hence, this study was conducted to assess the prevalence and determinants of scabies infestation in school-age children in Central Armachiho district, Northwest, Ethiopia. According to the result of this study, the overall prevalence of scabies among school-age children in the Central Armachiho district was 10.82%. This result is in line with studies done in Dabat district and Raya Alamata with a prevalence of 9.3% and 12.93% respectively [11, 30]. However, it is lower than the studies from Saudi Arabia (27.2%) [31], and Cameroon (17.8%). On the other hand, the prevalence of this study is higher than a study done in Egypt (4.4% [32]. This prevalence discrepancy might be attributed to family size variation, educational status variation, and might also be related to variation in sociodemographic characteristics of the study population, level of awareness, and health-seeking behavior across these populations.

This study indicates that the odds of having scabies among school-age children who had a contact history with confirmed scabies cases in the last two months were 5.28 times higher than those who had no contact history. This is supported by studies done in Dabat and Kacha Birra districts in Ethiopia, and Egypt [11, 33]. This might be due to contagious nature of scabies and one of the commonest ways of transmission is through body contact and mites easily pass from the infested person to normal one. S. scabiei var. canis and var. hominis mites were found to survive off the host for 24 to 36 hours under room conditions, this helped the mites to easily enter into human skin and abele to cause scabies infestation [14, 34].

According to this study, the odds of having scabies among school-age children that slept on the floor was 4.11 times higher among the school-age children who slept on the bed. This is supported by a study conducted in Ghana [35]. It might be due to the mites dropping to the ground and dust particles creating a favorable environment for the reproduction of mites and the individual who sleep on the floor can simply acquire scabies infestation [36].

In this study, the frequent use of detergents for hands and clothes was preventing scabies infestation significantly than only water users. This is consistent with a study conducted in Dabat, Ethiopia [11]. This might be due to the detergents will get rid of the scabies mites easily and it is important to remove immature mites from the skin so that the number of mites reduces the risk of transmission. Those school age children who use detergents frequently for showering and personal hygiene can easily prevent themselves from scabies infestation [19].

This study revealed that personal hygiene such as bedding/ cloth sharing; infrequent changing cloth, and not washing clothes for a prolonged period were also other predictors of scabies infestation according to this study. It was supported by the study conducted, Addet district, and Guna Begemder in Ethiopia and in Pakistan [37–39]. The reason is probably, the mites of scabies might exist on the bedding/ cloth and can stay out of human skin for up to 48 hours and can reside the parasite in the fomites like clothes, linen sheet and blankets that can have potential to transmit from infested to healthy once. less awareness about the importance of personal hygiene and poor personal hygiene might be a risk factor for the spread and transmission of scabies [10, 40].

School-age children who were not attended school were more likely to have scabies infestation as compared to those who are 5–8 grade. This is in line with a study conducted in Arbamich district, Ethiopia [10]. School-age children who were not attended school have less control and could have less awareness how to keep their personal hygiene and environmental sanitation which makes them more prone to be infested [41].

The odds of having scabies among school-age children who lived with displaced people were 3.47 times higher than school-age children who lived with non-displaced people. This study is supported by the study conducted in Sierra Leone [29]. The reason could be attributed to, in the displaced people certain environmental conditions, like overcrowding, poor personal hygiene, and poverty may be predominate. Subsequently, these conditions are conducive to the spread and increment of susceptibility to different skin problems like scabies infestation [23, 29].

## Strength and limitation of the study

### Strength of the study

- The study utilized large datasets representing the whole district,
- Data were recorded by well-trained data collectors under the close supervision of the supervisors

### Limitation of the study

- It could not establish a cause-effect relationship because of the cross-sectional nature of the study design.
- We didn’t use dermoscopy or skin scrapings/microscopy to diagnose scabies.

## Conclusion

The current study found nearly one out of ten school-age children had scabies in Central Armachiho district, Northwest Ethiopia. Not attending school, contact history with scabies confirmed patient of the last two months, not washing cloth for a prolonged period, infrequent changing clothes, cloth sharing, sleeping on the floor, living with internally displaced people and only use of water for handwashing practice were the factors that were significantly associated with scabies. Health care professionals better advise the parents/caregivers about the washing of clothing, changing clothes at least once per week, keeping physical contact with people who had confirmed scabies, promote detergent utilization for washing. Due to war in our country, there are large number of internally displaced people. Therefore, additional research is needed to assess the disease in the sites where displaced people reside, and having insight into the problem depth.

## Supporting information

S1 Table Indicates assessments of environmental, personal hygiene and sanitation factors. (DOCX)

## Abbreviations

HH: Head of House hold
Epi Info: Epidemiological Information
SPSS: Statistical Packages for Social Sciences
UOG: University of Gondar
WHO: World Health Organization
