# Supplementary material for: Prevalence and determinants of scabies among school-age children in Central Armachiho district, Northwest, Ethiopia
Source: PLoS One. 2022 Jun 14;17(6):e0269918. doi: 10.1371/journal.pone.0269918 (PMC9197047; doi:10.1371/journal.pone.0269918)
Supplement: S1 Table — (DOCX) [file pone.0269918.s001.docx]

| **Environmental characteristics and of the school age children** | | | | | | |
| --- | --- | --- | --- | --- | --- | --- |
| How far the health facility from household? | | _______________ | |  | |  |
| Place of sleep | | 1. On the floor 2. On bed | |  | |  |
| Have you ever had any skin contact with a person who had scabies (in the last two month)? | | 1.No 2. Yes | |  | |  |
| Household water consumption per head/day? | | _____________ | |  | |  |
| Source of water | | ____________ | |  | |  |
| How much far the water sources from household? | | ___________ | |  | |  |
| Have you/ family been in a village where active scabies endemic area in the last two months? | | 1.No 2. Yes | |  | |  |
| **Personal Sanitation and Hygiene practices** | | | | |  |  |
| How often do you take shower/bath? | 1. Never  2. Weekly  3. Every other week  4. Monthly  5. Quarterly  6. Twice a year  7. Once a year | |  | | |  |
| Do you wash your hands at critical time such as before &after eating food, before &after toilet? | 1.No 2.Yes | |  | | |  |
| What detergents do you use to take shower and hand washing? | 1.Water only  2.Water with Soap  3.Other | |  | | |  |
| Frequency of the Change of clothes | 1.A week and less than week  2. Above week | |  | | |  |
| Do you wash your clothes frequently? | 1.No 2. Yes | |  | | |  |
| If yes for Q.305, How often do you wash your clothes? | 1.Weekly  2.Every other week  3. Month  4. Every two month | |  | | |  |
| Are there any bedding/ cloth sharing commonly with others? (The last two month)? | 1.No 2. Yes | |  | | |  |
| Have you put on clothes (T –shirt or shirt, Shorts, pant) of someone who was diseased in the previous two months? | 1.No 2. Yes | |  | | |  |
| With whom do you sleep? | 1.Alone  2.Father  3. Mother  4. Brother  5.Sister  6. Friends  7.Other specify___ | |  | | |  |

**Supplementary file 1: indicates assessments of environmental, personal hygiene and sanitation factors.**
